# Fragile X syndrome and fragile X-associated disorders

**DOI:** 10.12688/f1000research.11885.1

**Published:** 2017-12-08

**Authors:** Akash Rajaratnam, Jasdeep Shergill, Maria Salcedo-Arellano, Wilmar Saldarriaga, Xianlai Duan, Randi Hagerman

**Affiliations:** 1 MIND Institute, UC Davis Health, Sacramento, CA, USA; 2 Department of Morphology and Obstetrics & Gynecology, Universidad del Valle, School of Medicine, Cali, Valle del Cauca, Colombia; 3 Department of Neurology, The Third Hospital of Changsha, Hunan Sheng, China; 4 Department of Pediatrics, University of California, Davis, School of Medicine, Sacramento, CA, USA

**Keywords:** whole exome sequencing, point mutations, copy number variants, FMR1 gene

## Abstract

Fragile X syndrome (FXS) is caused by a full mutation on the *FMR1* gene and a subsequent lack of FMRP, the protein product of *FMR1*. FMRP plays a key role in regulating the translation of many proteins involved in maintaining neuronal synaptic connections; its deficiency may result in a range of intellectual disabilities, social deficits, psychiatric problems, and dysmorphic physical features. A range of clinical involvement is also associated with the *FMR1* premutation, including fragile X-associated tremor ataxia syndrome, fragile X-associated primary ovarian insufficiency, psychiatric problems, hypertension, migraines, and autoimmune problems. Over the past few years, there have been a number of advances in our knowledge of FXS and fragile X-associated disorders, and each of these advances offers significant clinical implications. Among these developments are a better understanding of the clinical impact of the phenomenon known as mosaicism, the revelation that various types of mutations can cause FXS, and improvements in treatment for FXS.

## Introduction

Fragile X syndrome (FXS) is the most common inherited cause of intellectual disability (ID) and the most common single-gene cause of autism spectrum disorder (ASD). FXS arises from a full mutation repeat expansion (>200 CGG repeats in the 5′ untranslated region) in the *FMR1* gene on the X chromosome; this results in the methylation and subsequent silencing of the gene. Although FXS is the most well-known disorder caused by an *FMR1* mutation, there is also a spectrum of disorders associated with the *FMR1* premutation (55–200 CGG repeats) ^1^. Those with the full mutation have symptoms related to the absence or deficiency of FMRP, the protein encoded by the *FMR1* gene. These individuals are susceptible to global developmental delay, learning disabilities, and social and behavioral deficits. In contrast, those with the premutation allele have symptoms related to the elevated production of *FMR1* mRNA, leading to mRNA toxicity ^2^. Individuals with the premutation, especially males, are at risk for developing fragile X-associated tremor/ataxia syndrome (FXTAS), whereas females with the premutation have an increased likelihood of developing fragile X-associated primary ovarian insufficiency (FXPOI) before age 40 ^3^. Although global estimates for the frequency of both the full mutation and premutation exist, recent research has indicated that founder effects, as well as racial and ethnic differences, can significantly affect the risk of individuals in certain regions of the world. Thus, prevalence estimates may be more useful on a smaller, more regional scale ^4– 6^.

A robust clinical picture of full mutation FXS, as well as the variety of manifestations associated with the premutation, has existed for years; however, the improved understanding of mosaicism has begun to blur this picture. Mosaicism can refer to a condition in individuals who express both full mutation cells and premutation cells (size mosaicism) or individuals who have the full mutation but in whom only a portion of the full mutation alleles are methylated (methylation mosaicism). This may lead to unique cases, such as individuals with features of both FXS and FXTAS ^7^. Another discovery that has challenged previous understandings of FXS is that mutations other than full mutation repeat expansions have the ability to cause the disorder. The advent of more frequent whole exome sequencing (WES), whole genome sequencing (WGS) and microarray testing has led to the identification of different mutations such as point mutations, deletions, and duplications as causes of FXS. Moreover, these other mutations not only can cause FXS but also can result in partial FMRP functionality and lead to many subtly different phenotypes ^8^. There have also been rapid advances in the development of targeted treatments for patients with FXS over the past few years, as many animal studies as well as clinical trials have provided researchers with encouraging results. Certain treatments have been shown to reverse aspects of the neurobiological dysfunction in FXS; if used early in development, these treatments are likely to significantly improve the outcome of patients ^9, 10^.

## Clinical presentation of fragile X syndrome

Individuals with FXS present with varying degrees of cognitive impairment depending on sex and the level of FMRP produced ^11^. Patients producing higher levels of FMRP are typically less cognitively affected. Females with FXS, therefore, may present with a very wide range of clinical involvement due to differences in the activation ratio (AR). The AR refers to the proportion of normal *FMR1* alleles on the active X chromosome, which significantly impacts the amount of FMRP a female will produce ^12^. FMRP production also depends on CGG repeat number as well as the proportion of methylated full mutation alleles; therefore, individuals presenting with mosaicism are also likely to produce more FMRP. In turn, these individuals are typically less cognitively affected than non-mosaic patients with FXS ^13^.

**Figure 1.**
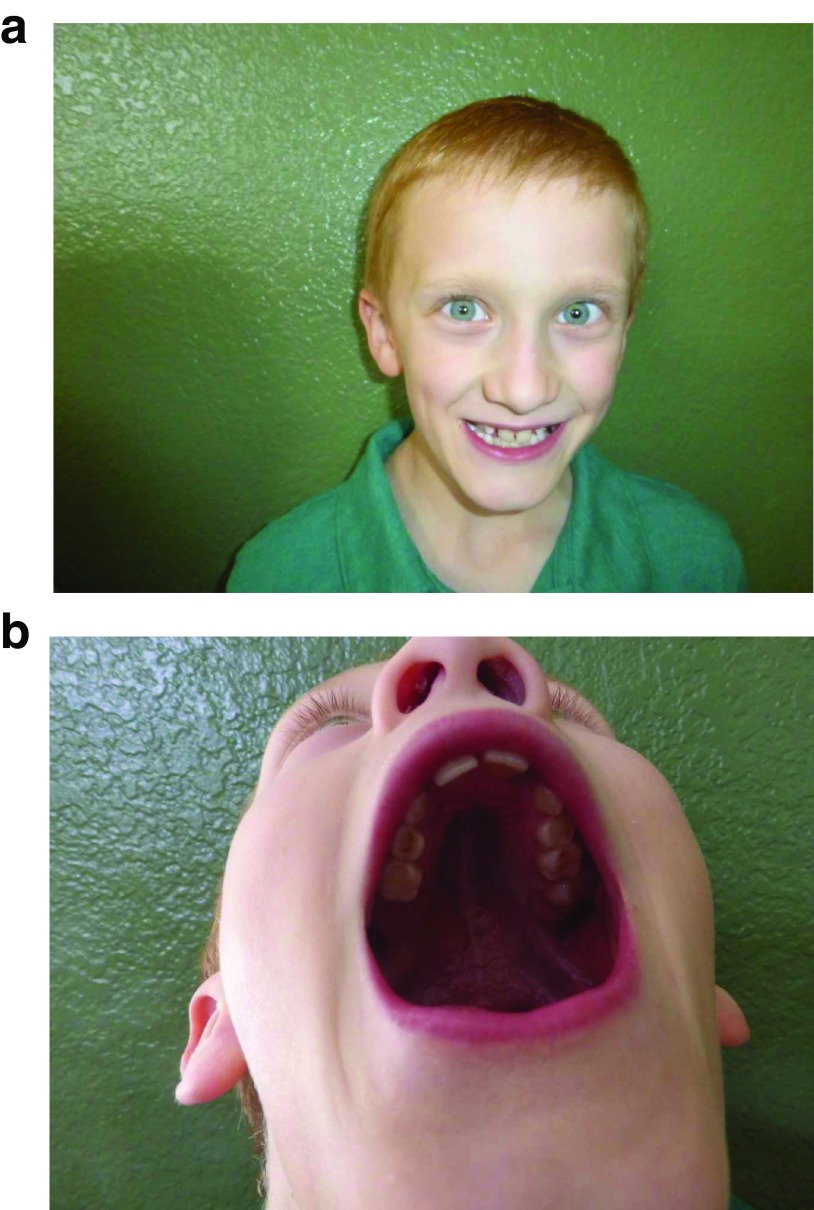
This 7-year-old boy with fragile X syndrome demonstrates a broad forehead ( **a**) and a high arched palate ( **b**) . However, he does not have a long face or prominent ears. He is a high-functioning individual with mosaicism, and DNA testing displays a band at 300 CGG repeats that is methylated as well as bands between 100 and 790 repeats that are unmethylated; overall, 30% of his alleles are unmethylated. He presents with a sequential IQ of 71 and a simultaneous IQ of 83. He has done well with treatment; sertraline has improved his anxiety symptoms, and a long-acting methylphenidate preparation has improved his attention-deficit hyperactivity disorder symptoms.

When FMRP levels are not significantly diminished, affected individuals may experience only modest socioemotional and learning deficits with relatively normal IQ levels. However, if FMRP production is severely decreased or fully silenced, moderate to severe cognitive dysfunction is likely to occur ^14^. In addition to ID, there are several physical features associated with FXS, such as a long face, broad forehead, high-arched palate, prominent ears, macrocephaly, and macroorchidism ( Figure 1 and Table 1) ^11^. Although not all individuals with FXS will have obviously dysmorphic features, roughly 80% of patients with FXS will present with at least one of these common characteristics ^14^.

**Table 1.** Clinical features of fragile X syndrome.

	Clinical characteristics	Prevalence
Physical	Long face Macrocephaly Prominent ears Prominent jaw Flat feet Joint hypermobility Macroorchidism	83%; occurs more commonly in adults 50–81% 75% 80%; occurs in adults only 29–69% 50–70%; occurs less commonly in adults 95%; occurs in adolescents and adults
Psychological	Attention-deficit hyperactivity disorder Anxiety Autism spectrum disorder	80% of boys and 40% of girls 58–86% 30–60%
Developmental	Intellectual disability Language deficits	85% of boys and 25–30% of girls ~100% of boys and 60–75% of girls
Other	Strabismus Recurrent otitis Gastrointestinal complaints Obesity Seizures	8–30% 47–75%; occurs in the first 5 years of life 31% 30–61% 15–20%

Clinical features of fragile X syndrome ^11, 14, 15^

**Figure 2.**
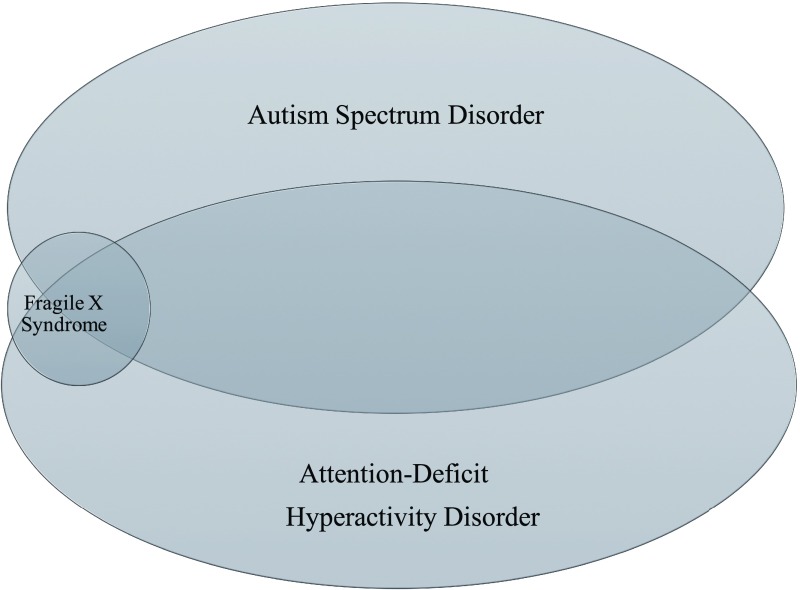
There is significant overlap between fragile X syndrome (FXS), autism spectrum disorder (ASD), and attention-deficit hyperactivity disorder (ADHD). Approximately 60% of all patients with FXS also meet criteria for ASD, although FXS accounts for only 2% to 6% of all cases of ASD. Furthermore, nearly 80% of children with FXS and 50% of children with ASD have co-occurring ADHD ^21, 22^.

A significant overlap exists between FXS and ASD. Monogenic disorders account for nearly one fifth of all diagnosed cases of ASD, and the most common of these monogenic causes is FXS ^16^. Many of the behavioral characteristics seen in individuals with FXS, such as impaired social communication, social anxiety, social gaze avoidance, and stereotypic behaviors, are the same traits seen in other causes of ASD. Moreover, a large portion of both individuals with FXS and individuals with ASD meet criteria for attention-deficit hyperactivity disorder ( Figure 2). Just as ASD is seen more often in males than females, males with FXS meet ASD criteria more frequently (60%) than do females (20%) ^17^. The overlap between FXS and ASD also has a molecular basis, as FMRP controls the translation of approximately 30% of the genes associated with ASD ^18^. These observations demonstrate that the underlying molecular etiologies of these disorders are intertwined, and targeted treatments of FXS may be helpful for other types of ASD.

## Clinical presentation of premutation disorders

Premutation expansions are associated with a variety of clinical manifestations, including psychiatric, developmental, and neurological problems. The premutation causes psychiatric problems, such as depression and anxiety, in approximately 50% of carriers. It also causes premature ovarian failure (menopause occurring before the age of 40) in a significant number of female carriers. This condition, known as FXPOI, occurs in 16% to 20% of female carriers; moreover, an additional 20% of carriers will experience menopause before the age of 45. Some clinical manifestations seen in those with the premutation are relatively common, even in individuals with normal *FMR1* alleles; however, their prevalence in premutation carriers is typically higher than their prevalence in the general population ( Table 2). The greatest clinical involvement associated with the premutation expansion results from the neurodegenerative phenotype known as FXTAS, which occurs in 40% of aging males and 16% of aging females with the premutation ^19^. FXTAS is a late-onset condition characterized by intention tremor, cerebellar ataxia leading to frequent falling, neuropathy, parkinsonian features, autonomic dysfunction, and cognitive decline ^20^. It is the most severe phenotype of premutation-associated disorders. FXTAS is also characterized by generalized brain atrophy and white matter disease in the middle cerebellar peduncle and the splenium of the corpus callosum. In addition, FXTAS is accompanied by the presence of intranuclear inclusions in both neurons and astrocytes in the central nervous system (CNS) as well as neurons in the peripheral nervous system ^19, 20^.

**Table 2.** Clinical involvement associated with the premutation.

Phenotype	Prevalence (premutation carriers versus general population)			
	Male carriers	Female carriers	Male non-carriers	Female non-carriers
FXTAS	40%	16%	N/A	N/A
Fragile X-associated primary ovarian insufficiency	N/A	16–20%	N/A	~1% (primary ovarian insufficiency)
Hypertension	57%	22%	~30%	~30%
Migraine	27%	54%	~12%	~20%
Neuropathy	62%	17%	<5%	<5%
Sleep apnea	32% with FXTAS	32% with FXTAS	~15%	~5%
Psychiatric problems	~50%	~50%	~ 3.6% (>45 years old)	~10.3% (>45 years old)

19, 20, 23– 28 Abbreviations: FXTAS, fragile X-associated tremor ataxia syndrome; N/A, not applicable.

Premutation disorders in extended family members are often identified when a proband is diagnosed with FXS, after which the proband’s mother is typically found to have the premutation. Moreover, if the grandfather of the proband also has the premutation, this means that all of the mother’s sisters will be obligate carriers as well. The *FMR1* premutation is known to be the most common genetic cause of primary ovarian insufficiency ^2^; thus, premutation disorders are now also being identified through OB-GYN offices, when fragile X DNA testing is ordered after seeing ovarian dysfunction. If a female is diagnosed with the premutation, her offspring have a 50% chance of inheriting a fragile X mutation; whether offspring inherit a premutation or a full mutation depends on the number of CGG repeats in the mother as well as on the number of AGG “anchors” present ^29^. AGG triplets, which typically interrupt CGG triplets after every nine or ten repeats, have been shown to decrease the likelihood of expansion to the full mutation when passed on to the next generation. Therefore, higher numbers of these AGG anchors in the maternal *FMR1* gene lower the risk of the full mutation in the offspring ^29^. If a male has the premutation, all of his daughters will also carry the premutation and are at risk of having children affected with FXS ^20^.

## Mosaicism

Two types of mosaicism exist: size mosaicism and methylation mosaicism. Size mosaicism refers to a condition in individuals carrying both cells with the premutation and cells with the full mutation in their blood. Furthermore, the ratio of premutation cells versus full mutation cells in the blood may differ when compared with other tissues, such as fibroblasts and brain tissue ^13, 30^. Methylation mosaicism refers to a condition in individuals who have the full mutation but only a portion of the cells containing full mutation alleles are methylated; methylation status may vary from tissue to tissue as well ^30^. The presence of either type (or both types) of mosaicism can blur the boundaries between the phenotypes of the premutation and the full mutation (FXS).

Individuals with methylation mosaicism produce more FMRP than individuals with fully methylated full mutation alleles ^31^. Additionally, CGG repeat numbers in the premutation range and FMRP expression are inversely related ^32^; therefore, individuals with size mosaicism who carry premutation alleles in addition to full mutation alleles will likely also produce more FMRP than non-mosaic individuals. Increased FMRP levels in FXS are associated with fewer clinical symptoms and correlate directly with IQ ^30^; thus, individuals with mosaicism tend to be higher-functioning. For example, one postmortem case study looked at multiple tissues of a high-functioning patient displaying both size mosaicism and methylation mosaicism. Only one region of the brain, the parietal lobe, had a methylated full mutation and silenced FMRP expression. However, FMRP was expressed in other parts of the brain, including the superior temporal cortex, frontal cortex, and hippocampus, which likely explains why this patient had only mild cognitive and behavioral deficits ^33^. Although mosaic individuals with FXS may be less cognitively impaired than non-mosaic individuals with FXS, they are more likely to develop FXTAS if their mRNA levels are elevated ^1, 20^. This can lead to an intriguing scenario known as the “double hit” phenomenon, in which an individual is affected by both lowered FMRP levels and elevated *FMR1* mRNA levels ^34^.

In 2013, Schneider and colleagues ^34^ documented two brothers in their 40s who both displayed this double hit of elevated *FMR1* mRNA levels and moderately decreased FMRP expression. The first brother, an individual with methylation mosaicism, presented with multiple physical features characteristic of FXS, such as macroorchidism, flat feet, and a prominent jaw. He was cognitively high-functioning but also displayed psychotic symptoms, including a history of bipolar 1 disorder. The second brother carried the premutation (118 CGG repeats); he experienced typical development but presented with a few physical features of FXS. Additionally, he presented with psychotic features associated with major depressive disorder. Another case report documented a 58-year-old male with size and methylation mosaicism and CGG repeats ranging from 20 to 800 ^30^. He presented with a slightly below average IQ and physical features of FXS. At age 50, he experienced neurodegenerative symptoms characteristic of FXTAS, including a worsening tremor and severe ataxia; he eventually experienced the cognitive decline typical of males with FXTAS. However, he was also an alcoholic, which may have increased CNS toxicity and exacerbated his FXTAS progression. Interestingly, he also met criteria for bipolar I disorder; taken together, these reports indicate that individuals with a double hit may be at an increased psychopathological risk. Additionally, these case studies challenge the traditionally clear distinction between FXS and premutation disorders and support the notion of a spectrum-based nature of disorders associated with *FMR1* mutations.

## Epidemiology

The reported rates of prevalence of FXS in the general population have been estimated to be approximately 1:5,000 males and 1:4,000 to 1:8,000 females ^35– 37^. However, the estimated prevalence of the mutation has varied because of the differing testing methodologies that have been used over time. Since *FMR1* testing began, the primary means of diagnosis has moved from cytogenetic testing for the presence of a folate-sensitive fragile site, to Southern blot analyses, to polymerase chain reaction (PCR)-based techniques. Additionally, wide variations in prevalence that are seen in different populations make global prevalence estimates difficult ^38, 39^.

To attempt to address these issues, Hunter and colleagues carried out a systematic literature review and meta-analysis of the prevalence of expanded *FMR1* alleles by using a random effects statistical model to analyze 54 epidemiological studies ^39^. After accounting for characteristics of the populations (that is, subjects with or without ID) and including only studies that used PCR and Southern blot techniques, obtaining screening data on over 90,000 females and 50,000 males; the determined rates of prevalence of the full mutation were 1:7,143 males and 1:11,111 females ^39^. Additionally, meta-analyses investigating the prevalence of premutation alleles among the general population have determined estimated frequencies of 1:150–300 females and 1:400–850 males ^35, 39, 40^. Although we are gaining a better understanding of the prevalence of repeat expansions, this is not the only type of mutation that can cause the disorder; a growing number of deletions and point mutations on *FMR1* have been identified in patients with FXS ^41, 42^. However, because repeat expansions are tested far more frequently than these other mutations, it is likely that the number of patients with FXS with non-repeat mutations has been underestimated. Ideally, this epidemiological shortcoming will be ameliorated through the increased use of WES and microarray testing going forward.

The frequency of expanded *FMR1* alleles varies globally because of both founder effects and racial differences in haplotypes that may predispose individuals in certain regions of the world to CGG expansions ^43^. Recently, the highest prevalence of expanded alleles has been reported in Ricuarte, a small town in Colombia ^4^. In Ricuarte, the rates of prevalence of the full mutation are 1:21 males and 1:49 females, whereas the frequencies of the premutation are 1:71 males and 1:28 females. This genetic cluster is likely a consequence of a strong founder effect from founding families that migrated from Spain. In sharp contrast to Ricuarte is Ireland, which has extremely low rates of prevalence of the full mutation: 1:10,619 males and 1:43,540 females. Researchers in Ireland speculate that a lineage-specific haplotype is responsible for this low incidence ^43^. China also has a relatively low reported incidence of FXS; however, owing to a gap between China and Western countries regarding FXS awareness, it is likely that a significant number of potential patients with FXS in China have been misdiagnosed or underdiagnosed ^6^. Other genetic clusters of fragile X mutations can be found in various parts of the world, including Indonesia, Finland, and the Spanish island of Mallorca ^5, 44, 45^. These observations suggest that the prevalence of FXS should be estimated separately in different countries or continental regions, as certain shared predispositions or strong instances of founder effects can lead to significantly different prevalence rates.

## Non-traditional ways of developing fragile X syndrome

FXS is almost always diagnosed through molecular testing for a CGG repeat expansion in the triplet repeat sequence of *FMR1*, as this is by far the most frequent cause of FXS ^8^. However, recent studies have implicated other mutations, such as point mutations and deletions, in those with FXS ^41, 42, 46^. There is nothing inherent about repeat expansions in the full mutation that cause the disorder; rather, it is the reduced FMRP production that leads to FXS ^8^. Thus, it is likely that any *FMR1* mutation that adversely affects the production of functional FMRP will lead to FXS. Indeed, a review of non-expansion *FMR1* mutations documented various deletions causing FXS; over 20 case reports documented FXS in patients who had normal CGG repeat numbers but had deletions of varying sizes in the triplet repeat sequence or flanking regions ^42^.

Though less common, numerous point mutations in *FMR1* have also been discovered in individuals with FXS and other developmental delays. In 2014, researchers documented FXS in a patient with a loss-of-function missense mutation (p.(Gly266Glu)) and a CGG repeat number of only 23 ^8^. A separate case study attributed a patient’s FXS to a loss-of-function nonsense mutation (p.(Ser27X); this patient’s CGG repeat number was just 29 ^46^. These case studies corroborate the notion that any mutation that interferes with the function of FMRP, not just full repeat expansions, can lead to FXS. Furthermore, two related studies sequenced the *FMR1* gene of hundreds of developmentally delayed males who did not meet diagnostic criteria for FXS; these mass sequencings revealed a total of three novel *FMR1* missense mutations ^41, 47^. Usually, developmentally delayed individuals with point mutations on *FMR1* have some of the features of FXS, although their phenotype may vary in some ways from that of the typical patient with FXS ^8, 48^.

## Targeted treatments

Significant advances have been made in the development of treatments for FXS. Animal models, including the *FMR1* knockout (KO) mouse and the Drosophila fly model, have provided researchers with several promising leads regarding effective pharmacological interventions for patients with FXS. In addition, many clinical trials have been carried out in the past few years and have been summarized in multiple comprehensive reviews ^49, 50^. Moreover, a number of these trials have led to medications that are now available for use by treating physicians. Minocycline, for example, is a semisynthetic tetracycline derivative that was first proven to be effective in improving anxiety and cognition in KO mice ^51^. A 3-month, double-blind controlled trial of minocycline in children and adolescents with FXS resulted in an improvement in global functioning as well as significant improvements in anxiety and mood-related behaviors ^52^. Lovastatin, a specific inhibitor of the cholesterol biosynthesis enzyme 3HMG-CoA reductase, has also shown promising results in KO mice; in these mice, lovastatin decreased excessive protein production and blocked epileptiform activity in the mice hippocampi ^9^. A controlled trial combining lovastatin treatment with parent-implemented language intervention (PILI) is currently being carried out at the UC Davis MIND Institute, investigating whether this combination of pharmacological and behavioral interventions will improve spoken language and behavior in children with FXS.

Another targeted treatment for FXS informed by animal studies is metformin, a drug that is currently approved by the US Food and Drug Administration for both obesity and type 2 diabetes. Drosophila fly models of FXS, as well as KO mice, have displayed metformin’s ability to improve defects in circadian rhythm, memory deficits, and social novelty ^53– 55^. Clinically, a recent open-label study saw anecdotal reports of improved behavior and language in children and adults with FXS ^56, 57^. Although initially metformin was hypothesized to be helpful in only those with the Prader-Willi phenotype (PWP) of FXS, associated with hyperphagia and severe obesity ^56^, this study suggests that patients without PWP or obesity may also benefit. This study demonstrates the need for a large controlled trial of metformin to see whether improvements in behavior, cognition, and language can be seen in children and adults with FXS.

The neurobiological overlap between ASD and FXS has led to the trial of a selective serotonin reuptake inhibitor (SSRI), specifically sertraline, to help alleviate the low serotonin levels seen in the CNS of those with ASD ^10, 58^. Metabolomic studies in various cases of ASD have demonstrated depressed levels of enzymes with the ability to metabolize tryptophan into serotonin, indicating that an SSRI may be helpful ^59^. An initial retrospective study of young children with FXS demonstrated a significantly improved receptive and expressive language trajectory in those on sertraline compared with those not on sertraline ^60^. A subsequent double-blind controlled trial of low-dose sertraline in young children with FXS found significant improvements in fine motor skills, visual reception, and the composite T score on the Mullen Scales of Early Development (MSEL). In a post-hoc assessment, the children with FXS together with ASD (60% of the study population) demonstrated significant improvement in expressive language on the MSEL when on sertraline ^61^.

Mavoglurant, an mGluR5 antagonist, has also been helpful in animal models, but efficacy has not been demonstrated in adolescents and adults with FXS ^62^. Similarly, arbaclofen, a GABAB agonist, has not shown efficacy in adults; however, outcome data for a limited number of measures in children look more promising ^63^. There may be a number of reasons for the failure of some trials to demonstrate efficacy. The outcome measures in many previous studies included only behavioral checklists, which are subject to parental bias ^62, 63^. Additionally, studies of arbaclofen and sertraline suggest that young children may respond better to targeted treatments than adults with FXS ^21, 61^. Addressing some of these concerns, a current trial of mavoglurant combined with PILI in young children with FXS is using novel outcome measures such as event-related potentials (ERPs), eye tracking methodology, and language sampling to better detect cognitive benefits and improvement in CNS function. Overall, a multitude of targeted treatments for FXS have provided researchers with encouraging results; however, much more research is needed before we can establish which interventions, or combinations of interventions, are the most effective for this population.

Whereas a significant amount of research has been dedicated toward treatments for FXS, research into targeted treatments for FXTAS and other premutation disorders is just beginning. A controlled trial of memantine, an *N*-methyl-D-aspartate (NMDA) receptor antagonist commonly used to treat Alzheimer’s disease, did not demonstrate improvements in tremor, ataxia, or executive function deficits in individuals with FXTAS ^64^. However, ERP studies demonstrated improvements in brain processing and attention in individuals with FXTAS when treatment was memantine versus placebo ^65, 66^. A more recent open-label study demonstrated that a weekly dose of intravenous allopregnanolone, a GABAA agonist, over the course of 12 weeks in patients with FXTAS resulted in improvements in both neuropsychological testing and neuropathy symptoms ^67^. As mentioned earlier, researchers are still in the early stages of finding effective targeted treatments for FXTAS and other premutation disorders, and there are many more clinical trials expected to come.

## Conclusions

Our understanding of FXS and other fragile X-associated disorders has grown significantly in recent years. It has become clear that disorders related to *FMR1* mutations are associated with a wide range of clinical presentations; there is a continuum of clinical involvement from premutation disorders into full mutation disorders due to both low FMRP and high *FMR1* mRNA. Some studies, such as reports indicating that high-functioning individuals with mosaicism are at risk for FXTAS, illustrate the ability of mosaicism to cloud the clinical picture of FXS and premutation-related phenotypes. Additionally, the number of detected deletions and point mutations in *FMR1* will continue to increase with the more widespread use of WES, WGS and the identification of these non-repeat mutations is further widening the spectrum of clinical involvement in FXS. Lastly, there is a promising outlook for effective targeted treatments; several medications have shown encouraging results in both animal models and clinical settings, and there are likely many more effective interventions to be found.

## Ethics

Written informed consent for the publication of the image found in Figure 1 was obtained from the child's parent.
